# Beyond Reason: Pandemic Resistance, Ontological Risk, and Behavioural Disintegration in Crisis Contexts

**DOI:** 10.1192/j.eurpsy.2026.11891

**Published:** 2026-07-31

**Authors:** C. Lazzari

**Affiliations:** Psychiatry, International Centre for Healthcare and Medical Education (ICHME), London, United Kingdom

## Abstract

**Introduction:**

Pandemics redefine more than epidemiological terrain—they fragment the ontological coherence of daily life. COVID-19 and HIV/AIDS both revealed that behaviour under crisis conditions cannot be fully explained by rationalist models or epidemiological logic. Instead, pandemic responses emerge from layered existential orientations shaped by perception, affect, and symbolic meaning.

**Objectives:**

To explore resistance to public health measures as a form of ontological dislocation and psychological enactment. This paper introduces the Quadruplex Helix framework as a model for understanding how unconscious drives, affective epistemologies, and social ontologies across relational levels shape dissemination resistance and critical incidents.

**Methods:**

A philosophical–psychoanalytic approach was adopted, integrating process ontology, phenomenology, and clinical literature. Textual analysis of case reports, ethnographic observations, and psychoanalytic theory (e.g., Samos Syndrome, irrationalism, solipsism) informed the development of a four-tier framework mapping behavioural responses across micro (individual), meso (dyadic), macro (peer), and mega (cultural) scales.

**Results:**

Resistance to preventive measures was observed not as ignorance but as an ambivalent negotiation of bodily boundaries and existential threat. Clinical and ethnographic findings revealed patterns of unconscious intent, such as self-endangerment, denial, and psychic conflict. The Quadruplex Helix delineated how different social strata produce distinctive behavioural expressions, including symbolic defiance, folie à deux dynamics, and cultural exceptionalism.

**Table 1.** Table 1. long description.

Helix Level	Domain	Key Focus	Behavioural Expression
**Micro**	Individual	Unconscious drives; psychic conflict; boundary dissolution	Self-endangerment, denial, symbolic refusal
**Meso**	Dyadic (interpersonal)	Relational distortion; affective contagion; folie à deux dynamics	Shared irrationalism, mutual reinforcement of resistant behaviours
**Macro**	Peer group	Collective enactment; group identity; affective epistemologies	Symbolic defiance, echo chambers, normative pushback
**Mega**	Cultural/societal	Mythic framing; national exceptionalism; ontological narratives	Cultural immunity myths, ideological resistance, existential reenactment

**Conclusions:**

Pandemic behaviours often emerge as meaning-making strategies under existential pressure, rather than as failures of knowledge. By reframing these actions through an ontologically informed lens, psychiatric models can better interpret disintegration of self–world boundaries, unconscious drives, and the phenomenology of risk. Future crisis interventions must recognise the symbolic, emotional, and relational architectures that shape public health behaviour.

**Disclosure of Interest:**

None Declared

## Long descriptions

### Table 1. Long description

The table consists of four columns: Helix Level, Domain, Key Focus, and Behavioural Expression.

* Row 1: Micro level. Domain is Individual. Key Focus includes unconscious drives, psychic conflict, and boundary dissolution. Behavioural Expression includes self-endangerment, denial, and symbolic refusal.

* Row 2: Meso level. Domain is Dyadic (interpersonal). Key Focus includes relational distortion, affective contagion, and folie à deux dynamics. Behavioural Expression includes shared irrationalism and mutual reinforcement of resistant behaviours.

* Row 3: Macro level. Domain is Peer group. Key Focus includes collective enactment, group identity, and affective epistemologies. Behavioural Expression includes symbolic defiance, echo chambers, and normative pushback.

* Row 4: Mega level. Domain is Cultural/societal. Key Focus includes mythic framing, national exceptionalism, and ontological narratives. Behavioural Expression includes cultural immunity myths, ideological resistance, and existential reenactment.

Navigate back to Table 1..

